# New Evidence of the Bidentate Binding Mode in 3-MBA Protected Gold Clusters: Analysis of Aqueous 13–18 kDa Gold-Thiolate Clusters by HPLC-ESI-MS Reveals Special Compositions Au*_n_*(3-MBA)*_p_*, (*n* = 48–67, *p* = 26–30)

**DOI:** 10.3390/nano9091303

**Published:** 2019-09-11

**Authors:** David M. Black, M. Mozammel Hoque, Germán Plascencia-Villa, Robert L. Whetten

**Affiliations:** 1 Department of Physics & Astronomy, University of Texas, San Antonio, TX 78249, USA; 2 Department of Biology, University of Texas, San Antonio, TX 78249, USA

**Keywords:** 3-MBA/Au MPCs, TEA-HFIP, ESI-MS, HPLC-MS, bidentate binding

## Abstract

Gold clusters protected by 3-MBA ligands (MBA = mercaptobenzoic acid, –SPhCO_2_H) have attracted recent interest due to their unusual structures and their advantageous ligand-exchange and bioconjugation properties. Azubel et al. first determined the core structure of an *Au_68_*-complex, which was estimated to have 32 ligands (3-MBA groups). To explain the exceptional structure-composition and reaction properties of this complex, and its larger homologs, Tero et al. proposed a “dynamic stabilization” via carboxyl O–H––Au interactions. Herein, we report the first results of an integrated liquid chromatography/mass spectrometer (LC/MS) analysis of unfractionated samples of gold/3-MBA clusters, spanning a narrow size range 13.4 to 18.1 kDa. Using high-throughput procedures adapted from bio-macromolecule analyses, we show that integrated capillary high performance liquid chromatography electrospray ionization mass spectrometer (HPLC-ESI-MS), based on aqueous-methanol mobile phases and ion-pairing reverse-phase chromatography, can separate *several* major components from the nanoclusters mixture that may be difficult to resolve by standard native gel electrophoresis due to their similar size and charge. For each component, one obtains a well-resolved mass spectrum, nearly free of adducts or signs of fragmentation. A consistent set of molecular mass determinations is calculated from detected charge-states tunable from 3− (or lower), to 2+ (or higher). One thus arrives at a series of new compositions (*n, p*) specific to the Au/3-MBA system. The smallest major component is assigned to the previously unknown (48, 26); the largest one is evidently (67, 30), vs. the anticipated (68, 32). Various explanations for this discrepancy are considered. A prospective is given for the *various* members of this novel series, along with a summary of the advantages and present limitations of the micro-scale integrated LC/MS approach in characterizing such metallic-core macro-molecules, and their derivatives.

## 1. Introduction

This work on the 3-MBA protected gold clusters, or cluster compounds, has, in brief, been motivated by the following circumstances: 

(i) A *Science* paper from 2014 identified a medium-sized (68, 32) 3-MBA gold cluster and determined its structure through an HREM-based statistical algorithm [1]. 

(ii) It has been proposed [2,3,4] that this ligand has a different ‘binding mode’ than its sister 4-MBA (pMBA), which is better understood thanks in particular to Vergara et al.’s recent subatomic resolution of the (146, 57) compound [5]. 

(iii) Previous attempts by the Tsukuda group to analyze these by ESI-MS are limited in scope, as the obtained spectra are “too broad” (unresolved), i.e., inadequate to establish the composition, e.g., is it truly Au_68_ (as the HREM reconstruction indicates)? What is the true ligand count (supplied by computational modeling)? 

(iv) According to our best evidence, the main component is (67, 30), rather than the previously published (68, 32), and the smaller main component is (48, 26). These unusual numbers, and indeed the entire graph of the observed composition number (*p* vs. *n*), are consistent with the theoretical and experimental (nuclear magnetic resonance, NMR) proposition of a special (bidentate) mode of 3-MBA binding. This trend-line (dependency) is in accord with the idea that as the size increases, the decreasing curvature (of the core’s surface) increases the propensity for the bidentate mode. Asymptotically, flat surfaces (self-assembled monolayers, SAMS) may be dominated by this binding mode.

Noble metal clusters, especially of gold and its intermetallic compounds, form highly stable complexes with thiolate and other pseudo-halide ligands. These are often called “monolayer protected clusters” (MPCs), because of their relation to the analogous self-assembled monolayers (SAMs) on planar or extended electrodes [6,7,8]. They have attracted special attention because of their nobility [9] (tolerance to air, moisture, and light; bio-compatibility, etc.); their facile modification via ligand exchange reactions [10,11,12]; a high-contrast detection, whether visual/optical or in X-ray and electron scattering [13]; and the fascination and potential utility of their strongly size-dependent optical, electrical and structure-bonding properties [14,15,16].

By now, much evidence has accumulated to suggest that many of these MPCs may be obtained in high yield as pure macromolecular substances of definite composition [17,18] and structure-bonding characteristics [19,20,21], as opposed to the more usual metal colloidal or nanoparticle [9] substances that often show heterogeneity. Such a proven structural uniformity of MPCs is essential to precision-intensive applications, as well as to all fundamental physicochemical understanding. The most compelling demonstrations are the cases of a total structure determination by single crystal X-ray [22] or electron diffraction methods [23], which for gold-thiolates have recently been extended to MPCs as large as Au_146_(pMBA)_57_ (aqueous) [5] and Au_279_(TBBT)_84_ (nonaqueous) [24].

Azubel et al. [1] determined the core structure of an *Au_68_*-complex via cryo-TEM, which was estimated to have 32 ligands (3-MBA groups). Tero et al. [4] proposed a “dynamic stabilization” mechanism via carboxyl O–H––Au interactions to explain its structure, composition and reaction properties, as well as those of its larger homologs [2,3].

Many reports have discussed the challenge of adequately characterizing samples of novel MPCs, particularly in the early stages of identifying the main compounds or components of a mixture, as discussed elsewhere [25,26]. Our approach here has been to adapt a method—electrospray ionization (ESI)-coupled high performance liquid chromatography mass spectrometry (HPLC-MS)— established earlier for bio-macromolecules of a similar size (or mass) and surface chemistry as the MPCs under investigation [27]. Specifically, the larger Au/MBA clusters have many (~24–60) acid-terminated ligands [3], and so are presumed to exist in an aqueous solution at a normal (or higher) pH as poly-anions (plus respective counter-cations). For this case, a long experience with oligonucleotides (DNA or RNA), composed of a similar number, ~24–60 base-sugar-phosphate repeats), seems most instructive. 

Our aims in the present work have been (i) to determine whether the unusual solution-phase characteristics of the Au/3-MBA clusters will permit them to yield to be analyzed by the ESI-coupled LC-MS methods that have recently improved the analysis of Au-pMBA clusters ranging from small oligomers and clusters (25, 18) and (36, 24) to the larger species (102, 44), (130, 50) and (144, 60) [27]; (ii) to examine whether ion-pairing agents will work similarly to enable both high-resolution LC separations and reduced-fragmentation ESI-ToF (time-of-flight) mass spectra; (iii) to provide some insight into the powerful selection principles underlying the results in refs. [1,2,3,4]; (iv) to search for minor or hidden components (new compositions) as semi-stable or transition MPCs; and (v) to provide additional evidence pertaining to the ‘bidentate’ or dynamical carboxyl-gold interactions described in reference [4].

Herein, we report the first results of an ESI-coupled LC-MS analysis of unfractionated samples of Au/3-MBA clusters that span a narrow mass range, 13.4–18.2 kDa. Using procedures adapted from oligonucleotide analyses, we show that integrated capillary HPLC-ESI-MS, based on aqueous-methanol mobile phases and ion-pairing reverse-phase chromatography, can separate *at least two* major components (and several minor ones) that are present in all sources. For each component, a well-resolved mass spectrum, nearly free of adducts or signs of fragmentation, allows the determination of a consistent assignment of molecular masses, as calculated from detected charge-states tunable from 3− (or lower), to 2+ (or higher). One thus arrives at a set of proposed compositions (*n*, *p*), as characteristic of the Au/3-MBA system. The smaller major component is assigned to the previously unknown (48, 26); the larger one is assigned to (67, 30), vs. the anticipated (68, 32). 

## 2. Materials and Methods

### 2.1. Synthesis

The size-uniform samples prepared at the University of Texas at San Antonio by Germán Placencia-Villa (*GPV*) for this work are synthesized according to a modified Brust-Schiffrin approach described elsewhere [1]. In brief, a stirred solution of 3:1 molar ratio of 3-MBA—HAuCl_4_ (Sigma Aldrich, Saint Louis, MO, USA) was allowed to equilibrate for 16 h under basic conditions in 30% methanol (Fisher Scientific, Hampton, NH, USA) solution prior to the cluster-forming reduction reaction initiated by the addition of sodium borohydride (Sigma Aldrich, Saint Louis, MO, USA). This altered method has been shown to produce uniformly sized clusters, as opposed to the production of many discretely sized particles. 

### 2.2. 3-MBA/Au System Characterization

The characterization of molecular nanoparticle preparations is carried out by a variety of methods for the characterization of the system of interest. Size-exclusion [28], gel-permeation [29], thin-layer chromatography [30], gel-electrophoresis [31], reversed-phase [32], and hydrophobic interaction [33] liquid chromatography has all been extensively used as essential analytical tools for the characterization of such nanoclusters. These methods separate the various components of a mixture according to one or more physical and/or chemical attributes, including differences in size, polarity, hydrophobic character, and electrophoretic mobility (related the size-to-charge ratio). Ion-pairing can be combined with reversed-phase LC for the analysis of acidic and basic clusters [34]. Separation methods may be used alone for sample fractionation or in conjunction with various detectors. 

The analysis of nanoclusters through these methods is only possible for those samples exhibiting a certain degree of modal- or multi-modal distribution—with each mode showing a minimal variance. Samples that exhibit a continual distribution, as is the case of nanoparticles exceeding an approximately 3-nm core diameter, are not amenable to LC or MS analysis. ‘Magic-number’ nanocluster preparations are good candidates for a characterization by liquid chromatography and mass spectrometry because these clusters form in a multi-modal fashion, with only a few compositions exhibiting a high degree of stability. The LC-MS data acquired from these samples may be used to assign a specific cluster identity as well as for the semi-quantitative determination of each of the components present in a sample. Aqueous nanoparticles, like those investigated here, are of interest because of their potential application in medical and life sciences [2,3].

#### 2.2.1. Coupled Chromatography—ESI-MS

In the present work, efforts were focused to determine whether the 3-MBA/Au systems were amenable to analysis by HPLC-ESI-MS in the same way as previously observed for the analogous 4-MBA/Au systems (aka p-MBA/Au). Specifically of interest was the possibility of ion-pairing with triethylammonium cations (TEAH+) [27] for the retention and separation of these poly-acid clusters via reversed phase chromatography. Additionally, of interest was an understanding on the effectiveness of this ion-pairing strategy for electrospray ionization (ESI) and if the necessary conditions could be implemented for a supported determination of cluster compositions with some degree of clarity by minimizing fragmentation. The successful implementation of HPLC-MS to these systems may help reveal ‘hidden components’ [35]—not otherwise known or detectable by native PAGE gel-electrophoresis. Any evidence to support or refute the proposed ‘bidentate’ bonding (H-bonding of carboxyl to Au) is also of interest in these studies. 

Although gel-electrophoresis is a standard technique for the analysis of nanoparticle preparations, it is a relatively coarse size separation method. An exact determination of size and uniformity requires confirmation by a secondary analytical technique, since it is possible for the components having different sizes, shapes, or charges to share the same, or similar, electrophoretic mobilities. 

#### 2.2.2. HP-LC–ESI-MS Sample Preparation

The obtained Au/3-MBA samples were either re-dispersed or diluted—if a solid or solution, respectively—approximately 10× in the appropriate solution. LC separation was performed with coupled electrospray time-of-flight mass spectrometry detection (ToF-MS). The separations were carried out on a C_18_ stationary phase using gradient methods, whereby the initial mobile phase composition was replaced with a higher organic concentration in a linear fashion over a period of twenty minutes. The instrumental procedures, i.e., mass spectrometer, HPLC columns used in this work, are described elsewhere [36] in the HPLC-MS and UV−Vis Method Conditions section. The mobile phases were prepared containing 400 mM hexfluoroisopropanol (HFIP)-15 mM triethylamine (TEA), TEA-HFIP *or* 10 mM triethylammonium acetate (TEAA) in ddH_2_O (mobile phase A) and methanol (mobile phase B). The separation behavior of the nanoclusters predominantly depends on the selected combination of the stationary phase, mobile phase, gradient, and mobile phase modifier. Starting from the conditions used to obtain a satisfactory separation and ionization of our previous report of larger p-MBA/Au MPCs [27], the gradient and modifier selection were varied to find the conditions for the satisfactory separations for this current 3-MBA (aka m-MBA)/Au MPCs work. In this work, 10–40% MeOH (mobile phase B) gradient over 20 min at ambient temperature were used for the efficient separation of the clusters, though the selection of the modifier (ion-paring agent) plays the vital rules as demonstrated in our results. The near-baseline separation of the various components is crucial for providing a differentiation and correlation between the various MS signals observed, which aids the MS interpretation and reduces the possibilities for ion-suppression artifacts. The gradient method can be adapted to produce a greater separation between components, and the mobile phase modifier is essential for a good chromatographic performance compatible with acceptable electrospray ionization. Solution phase ion-pairing effectively neutralizes the MBA’s carboxylate (–COO^−^) group by association with TEAH^+^, enhancing the interaction of the mercaptobenzoic acid ligands with the C_18_ stationary phase. 

## 3. Results

Recent reports have demonstrated the possibility of producing uniformly-sized batches of 3-mercaptobenzoic acid (MBA) protected nanoparticles [1,2,3,4]. Smaller nanoparticles, or nanoclusters, are noteworthy for their interesting properties, and because certain stoichiometries (i.e., gold-to-ligand ratios) form in abundance due to their relatively higher thermodynamic stability [4,37]. This phenomenon makes it possible to produce specific nanomolecular particles in abundance. However, because there exist various “magic-number” sizes (e.g., Au_25_, Au_38_, Au_68_, Au_102_, Au_144_, etc.), nanocluster preparations may still exhibit heterogeneity or mixtures varying from one batch to the next. These improved synthetic procedures make it possible to produce size-focused preparations of nanoparticles, thus enabling the production of a higher-quality product. 

Synthetic procedures such as these, in tandem with analytical methods that can be used to characterize these preparations, may provide the needed capabilities for the development of various nanoparticle applications. The 3-MBA/Au systems demonstrate ‘certain advantages’ over other thiolates (organic or hydrophobic) for the purposes of ligand exchange and conjugation, as well as for bio-applications.

**Figure 1:**
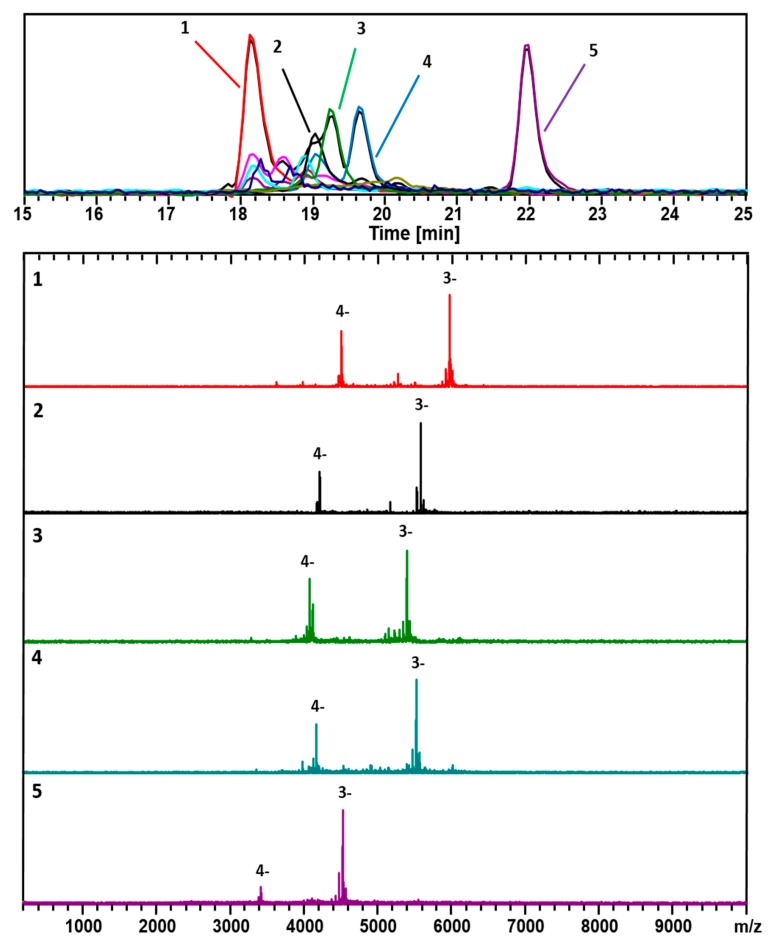
ESI-coupled LC-MS analysis of Au/3-MBA clusters from the Azubel-preparation. Detection is set for negative ions, under conditions that generate mainly 3− and 4− charge states. The top frame shows the chromatograms, i.e., the base peak chromatogram (*m*/*z* 100–10,000), and an extracted-ion chromatogram (EIC) for each identified component. The color-coded EIC chromatographic peaks track with the coded and numbered mass spectra listed herein with compositions assigned as follows: (1, Red) (67, 30), 17.8 kDa; (2, Black) (60, 31), 16.6 kDa; (3, Green) (58, 30), 16.0 kDa; (4, Blue) (60, 30), 16.4 kDa; and (5, Purple) (48, 26), 13.4 kDa. The fine-structure of the (67, 30)^3−^ complexes is presented in Figure S1.

Figure 1 shows negative-ionization (-ESI) LC-MS mode data acquired following the sample (prepared by the size-uniform synthesis procedure) provided by M. Azubel, as prepared at Stanford University. Two dominant components are readily identified: (67, 30; 17.8 kDa) and (48, 26; 13.4 kDa). These appear at the short (long) retention times and high (low) mass ends of the spectrum. 

**Figure 2:**
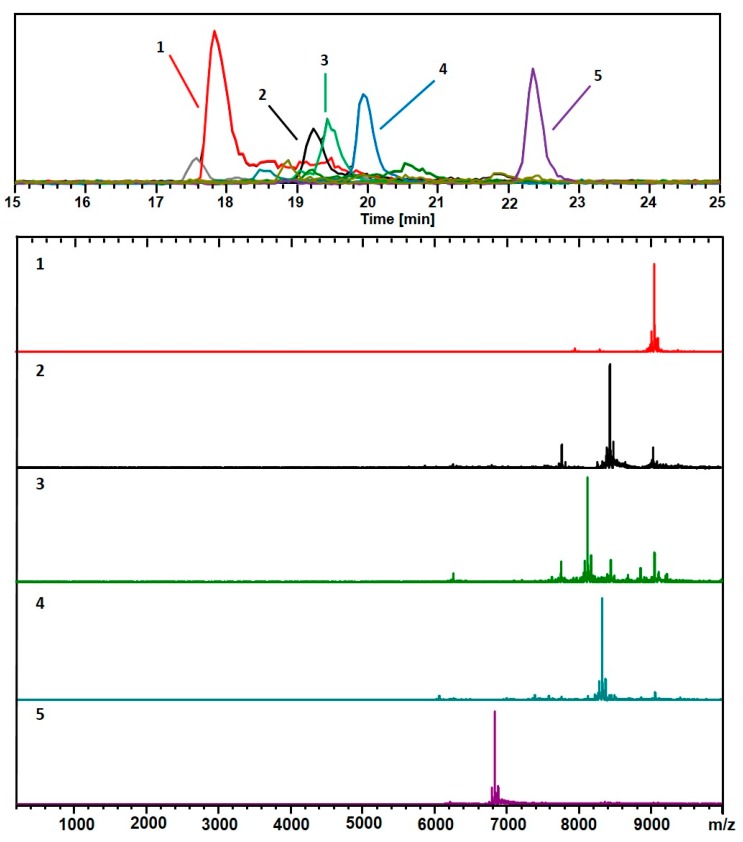
As in Figure 1, but with a positive (ESI+) mode for detection. This analysis shows mainly 2+ charge-states. The black trace corresponds to the base peak chromatogram (*m*/*z* 100–10,000). The color-coded EIC chromatographic peaks track with the coded and numbered mass spectra listed herein, with compositions assigned as follows: (1, Red) (67, 30), 18.1 kDa; (2, Black) (60, 31), 16.9 kDa; (3, Green) (58, 30), 16.2 kDa; (4, Blue) (60, 30), 16.6 kDa; and (5, Purple) (48, 26), 13.6 kDa. The fine structures of the (67, 30)^2+^ complexes are presented in Figure S2.

Figure 2 shows the results from the analysis of the same sample, obtained in the positive-ionization (+ESI) LC-MS mode. The mass spectra are shown for each of the 5 major components: (67, 30; 18.1 kDa), (60, 31; 16.9 kDa), (58, 30; 16.2 kDa), (60, 30; 16.6 kDa), and (48, 26; 13.6 kDa). These are also considered when assigning the compositions listed in Figure 1. In both cases, 10 mM TEAA was used as the ion-pairing agent to facilitate the ionization process. 

**Figure 3:**
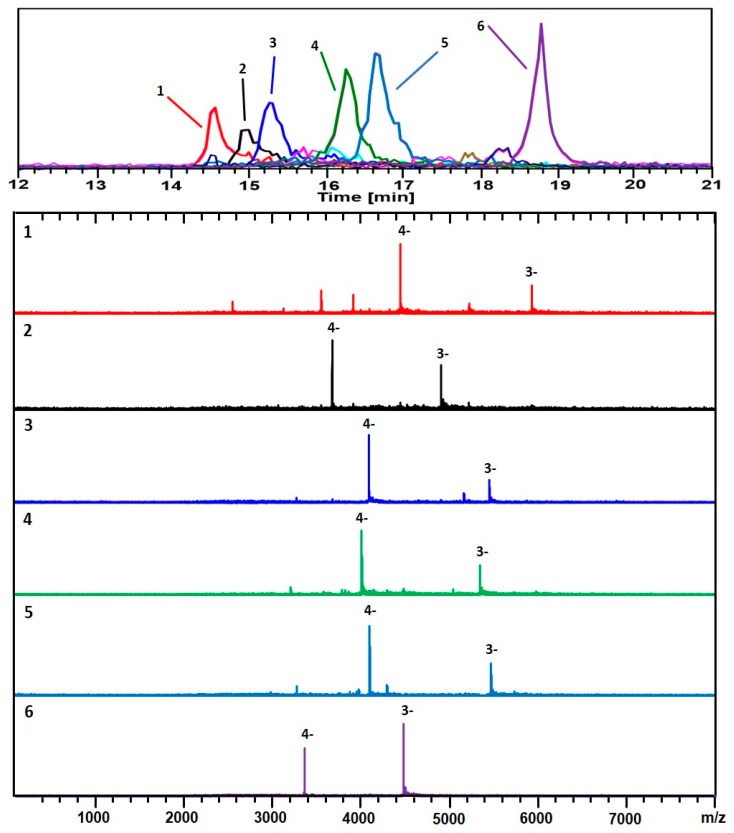
The analysis of a second preparation (*GPV*) of Au/3-MBA clusters. Negative ionization mode (-ESI) detection shows mainly 3− & 4− charge-states. The black trace corresponds to the base peak chromatogram (*m*/*z* 100–8,000). The color-coded EIC chromatographic peaks track with the coded and numbered mass spectra listed herein, with compositions assigned as follows: (1, Red) (67, 30), 17.8 kDa; (2, Black) (53, 28), 14.7 kDa; (3, Blue) (59, 31), 16.3 kDa; (4, Green) (58, 30), 16.0 kDa; (5, Light Blue) (60, 30), 16.4 kDa; and (6, Purple) (48, 26), 13.4 kDa. For the singly charged (z = 1−) of the same sample, see Figures S3 and S4. The polyacrylamide gel-electrophoresis (PAGE) analysis and corresponding HPLC-ESI-MS chromatogram are presented in Figure S4.

**Figure 4:**
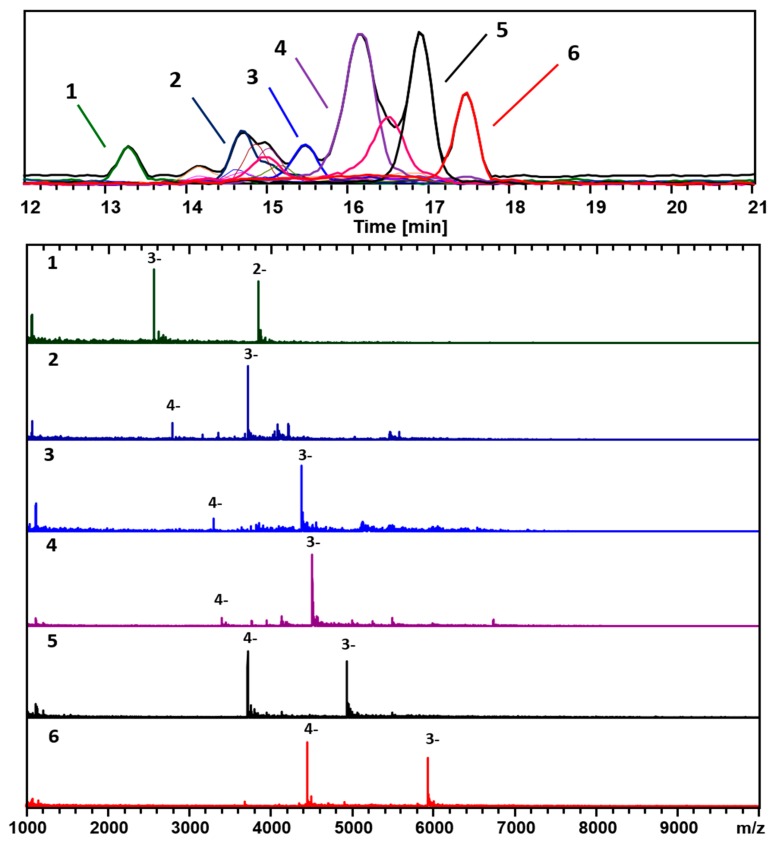
The analysis of the second preparation of 3-MBA clusters using the TEA–HFIP mobile phase buffer composition. The negative ionization mode was used for the analysis, mainly 3− and 4− charge-states. The black trace corresponds to the base peak chromatogram (*m*/*z* 1000–10,000). The color-coded EIC chromatographic peaks track with coded and numbered mass spectra listed, and the compositions are assigned as follows: (1, Dark green) (25, 18), 7.7 kDa; (2, Dark Blue) (38, 24), 11.1 kDa; (3, Blue) (46, 26), 13.0 kDa; (4, Purple) (48, 26), 13.4 kDa; (5, Black) (53, 28), 14.7 kDa; and (6, Red) (67, 30), 17.8 kDa.

Figure 3 and Figure 4 show two analyses with two different ion-pairing agents TEAA and TEA-HFIP, respectively of a separate preparation (*GPV*) of 3-MBA clusters. 

Besides the main components, (67, 30; 17.8 kDa) and (48, 26; 13.4 kDa), identified in Figure 1 and Figure 2 (*Azubel*’s sample), several other minor ones, (25, 18; 7.7 kDa), (38, 24; 11.1 kDa), are identified with our ESI-MS method, especially at a smaller mass. Figure 4 shows results for the same sample analyzed using a combination of a more volatile weak acid HFIP than acetic, and TEA. Interestingly, while the components observed in each analysis are essentially identical, the order of elution is significantly altered for the two modifiers. The TEAA modifier produces a chromatography whereby the larger clusters generally elute first, followed by smaller ones. The TEA-HFIP reverses this general trend, so that smaller clusters elute first, followed by larger ones.

**Figure 5:**
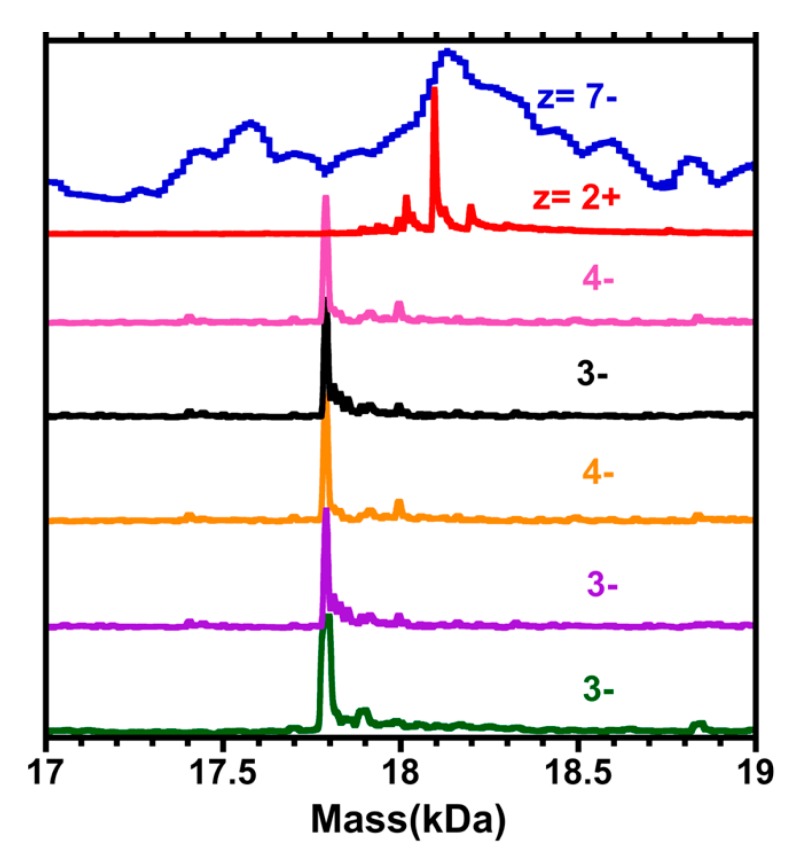
A comparison of the deconvoluted mass spectra in the region of the 17.8 kDa compound, putatively “Au_68_(3-MBA)_32_”, vs. the ESI-MS of reference [1] (blue curve at top). Selected portions of the ESI mass spectra of gold cluster samples are depicted, in which the independent variable has been converted from the (*m*/*z*) scale to the total mass (kDa), using the charge (z) assignments indicated in Figure 2 (red), Figure 4 (pink and purple), Figure 1 (orange and black), and Figure 3 (dark green). Note that in the case of the positive ion mode (z = 2+), the peak is shifted higher by ~ +0.3 kDa, consistent with three (3) TEAH^+^ adducts, to the (67,30)^1−^ complex. [Mass of TEAH^+^ = 101 Da.].

Figure 5 contains a comparison among the mass spectra above (Figure 1, Figure 2, Figure 3 and Figure 4), as they pertain to the putative “Au_68_(3-MBA)_32_” compound (calculated mass of 18.3 kDa), and also to an extract from the mass spectrum provided in reference [1]. In negative-ion detection, as appropriate to polyacids, the evidence all points toward 17.8 kDa, the mass of (67, 30). In positive-ion detection, where TEAH+ adducts provide the charge, the mass of 18.1 kDa also agrees with (67,30), assuming triple-adduction, i.e., 3 TEAH+, in which case the (67, 30) complex carries an intrinsic (core) charge of 1−.

**Figure 6:**
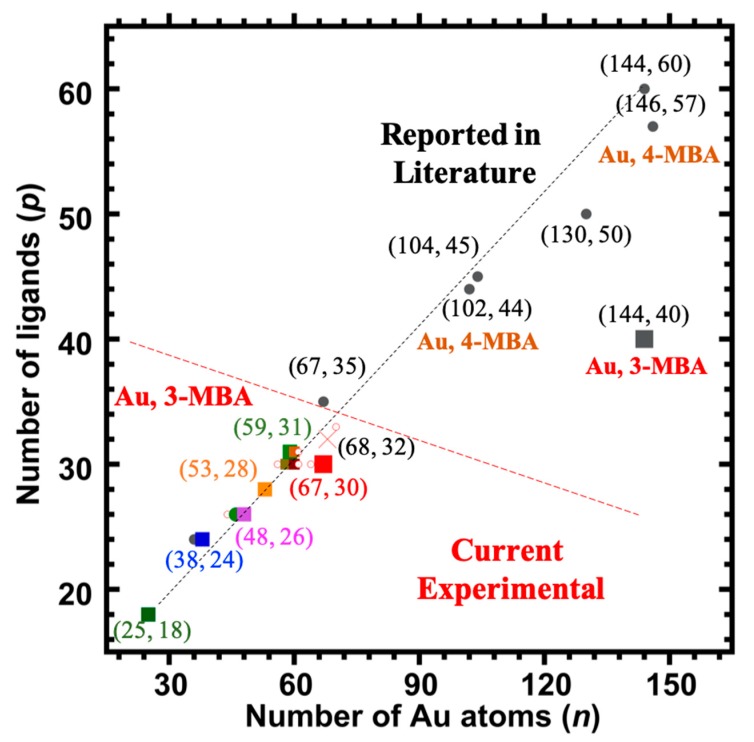
The number of ligands (*p*) vs. the number of Au atoms (*n*) found in this work on the Au*_n_*(3-MBA)*_p_*, (*n* = 48–67*, p* = 26–30) and reported in the literature are shown to see the trends. Legend: symbols designate whether the component identified was detected prominently (colored large square) and for both samples under all ESI-coupled LC/MS conditions, or not (small hollow red circle); a red cross represents the expected (68, 32) clusters, a grey large square represents 3-MBA thiolated larger (144, 40) clusters, whereas small grey circles (filled) represent reported different compositions of other thiolated clusters, for example 4-MBA (a sister molecule of 3-MBA) thiolated Au*_146_*(4-MBA)*_57_*.

Figure 6 shows plots of the various chemical compositions observed for each of the different samples analyzed here. Although a number of different compositions were observed for each sample, a clear difference between the size-uniformity of the two samples can be observed. When a long equilibration prior to reduction is carried out, a much narrower range of cluster sizes is formed. If the procedure is varied––even slightly––to reduce this time period, a wider range of cluster sizes is formed, and this is supported by the recent reported “captamino”, a base side, thiolated gold clusters in the size range of 25–144 numbers of Au, or even larger ones [17,18]. In Figure 3, cluster compositions ranging from (47, 26) to (71, 34) are observed; whereas in Figure 4, compositions ranging from (25, 18) to (67, 30) are observed.

## 4. Discussion

### 4.1. General Remarks

As mentioned in the Introduction, our general objective in this research has been to advance the analytical chemistry of thiolate-protected gold clusters. Specifically, we have aimed to adapt the standard HPLC-ESI-MS methodology, as applied for example to oligonucleotides, which are acidic (poly-anionic), developing optimized strategies for gold clusters protected by a monolayer of thiolate ligands with terminal (solution-exposed) acidic groups. The recent progress reported by Black et al. includes the HPLC-ESI-MS identification of a long series of Au-pMBA clusters as large as (146, 57) [5], or as small as (25, 18) and (36, 24). Through the use of a suitable ion-pairing agent (TEA+), well resolved mass spectra could be obtained under gentler conditions (to reduce poly-anion fragmentation in electrospray ionization) and nearly free from alkali-ion and solvent adducts [27]. In a related work, silver-lipoate clusters (29, 12)^3−^ have been effectively resolved, where lipoate acts as a bidentate (di-thiolate) ligand with a terminal carboxylate (“thioctic acid”) [36,38].

### 4.2. Contrasting 3-MBA (or Meta-MBA) and 4-MBA (Aka Para-MBA)

In turning from the 4-MBA (pMBA) to 3-MBA (or “mMBA”) ligands, one faces a more challenging analytical situation, as described most recently in a 2017 *ACS Nano* report by Tero et al. [4], as well as in the earlier reports of Azubel et al. [1,2,3], dating to the 2014 *Science* article:

- There has been no total-structure determination of any 3-MBA protected gold clusters.
- There has been no adequately resolved ESI-MS identification of any of these: no composition-determination by any standard analytical method.
- Electron microscopy (or diffraction) provides the gold structure and atom count, in both (2) reported cases. (Ligands/S-atoms are not located by this method). Models are then constructed, which include the ligands, and these are tested (refined) by DFT computations.
- The compositions arrived at by these procedures, (68, 32) and (144, ~40), are respectively distinctly and strikingly different from those determined previously for aliphatic ligands, i.e., (67, 35) and (144, 60), or from the more directly relevant water-soluble aromatic pMBA ligand (146, 57). [Figure 6 presents these compositions in a graphical format.]

In practice, (via the same HPLC-ESI-MS optimized procedures) we were able to readily obtain clear results on the samples believed to be dominated by the (68, 32), but not on the samples labeled as larger compounds (144, ~40). This is not particularly surprising, for large polyanionic assemblies have a reputation for presenting a difficult ESI-MS analysis. For the same reason, the presence of readily detectable smaller components, such as the major one assigned to (48, 26), or even the minor one (25, 18) in one instance, is unsurprising, as their signal levels may be disproportionate to their concentration in solution.

Perhaps the major positive result of our work is the greatly improved (vs. 2014 Science report^1^) quality of ESI mass spectra (Figure 5) that led us to identify (67, 30) as the composition of the compound previously assigned to (68, 32). This is only a minor difference, amounting to a single gold atom and two (2) 3-MBA ligands, but could suggest a reinterpretation of its structure and bonding.

However, one should note that this suggested revision (reducing the ligand-count to 30 from 32) only serves to increase its distinctiveness, as compared to the reference (aliphatic) case, i.e., (67, 35) vs. (67, 30). Now, the ‘ligand deficiency’ (below) is five (5) rather than two (2), as indicated in Figure 6. The other components, and specifically the one identified as (48, 26), may also be interpreted within this same context of ‘ligand deficiency’. Figure 6 shows that, for gold clusters protected by thiophenol-class thiolates, the important compositions (36, 24) and (44, 28) lie on a distinct ‘curve’. Yet the smallest (minor) compound identified here as (25, 18) is the same regardless of the thiolate.

Below, we suggest how the ‘dynamical stabilization’ model—a form of bidentate ligation—of Tero et al. [4] can be generalized, from the two cases {(68, 32), (144, ~40)} investigated by them, to account for the entire range of compositions identified and presented in Figure 6. The dynamic-stabilization model (DSM) [4] was reported to account for the optical (FTIR) spectra as well as other analytical observations, in a way that also explains the ligand-count deficiency and the lability of these two compounds when exposed to other (non-3-MBA) thiolates in solution. In particular, the basis for this model is described as follows:

- In the carbonyl (C=O) stretching region, the vibrational FTIR spectra show “distinct peak[s] around 1730 cm^−1^, observable only in 3-MBA-passivated clusters, and interpreted as the signal of the O=C−OH···Au interaction.” [4].
- Molecular dynamic (MD) simulations were based on structure models for each cluster. “Visual inspection of MD trajectories revealed several weak interactions in the ligand layer and at the ligand−gold interface, such as formation of inter-ligand hydrogen bonds, inter-ligand π stacking (aromatic contacts), π−Au interaction where the aromatic ring lies “flat” on the gold core, and hydrogen bonding-like O=C−OH···Au interaction when the hydroxyl group is rotated toward the gold core.” “*We thus assigned the highest frequency observed for both Au_144_(3-MBA)∼_40_ and Au_68_(3-MBA)_32_ to the O=C−OH···Au interaction visualized* … This interaction at the ligand−metal interface has not been reported before for any thiolate protected gold nanocluster.” [4].

In the report, we have referred to any such interaction involving a second functional group (other than thiolate sulfur) as a “bidentate” bonding mode, whether the carboxyl group is protonated or de-protonated (as is more typical in solution-phase conditions, pH neutral or > 7). 

First, the ligand-deficiency count, which ranges from 15–20 in the case of (144, ~40), amounts to five (~5) for (67, 30), to perhaps a couple (2) in the case of (48, 26), and finally to zero (0) in (25, 18), is taken to represent *the number of ligands bound in a bidentate fashion*. It is represented as a fraction of the whole: In the extreme case of (144, ~40), more than one-third of the ligands are bound in the bidentate mode. In the special case of (67, 30), one-sixth (5/30) are bidentate, and for (48, 26) only one-twelfth (2/26) are so indicated. 

Second, as usual the key step is to relate these fractions to the estimated curvature (1/R) of the structure as measured at its surface, where R is the radial distance at which the Au–S or Au–X bonds lie. A high curvature removes the driving force for bidentate coordination, because the steric constraints are greatly reduced (the position meta to sulfur should be well exposed to the solvent and counter-cation).

For now, we leave this as a semiquantitative argument suitable for guiding further work on both the larger, or previously identified compounds, as well as the ones newly identified in this report. The need for this was well predicted in the closing remarks of reference [4]: “Several currently unknown compositions and sizes of 3-MBA-protected gold nanoclusters will undoubtedly be found by variations of the known syntheses, which will open unexplored possibilities for applications of these materials in biolabeling, catalyzing biochemical reactions, imaging, detection, and theranostics.” 

## Supplementary Materials

The following are available online at https://www.mdpi.com/2079-4991/9/9/1303/s1; Figures S1–S4, fine-structure in the electrospray negative ionization mode mass spectrometric analysis of the (67, 30), complex (67, 30)^z−^ in solution, electrospray positive ionization (ESI+) mass spectrometric analysis of the component identified as (67, 30), by HPLC-ESI-MS as in Figure 2, ESI-MS Analysis of *GPV* sample preparation, under conditions wherein mainly the singly charged (z = 1−) ions are detected, and the polyacrylamide gel electrophoresis (PAGE) and HPLC analyses of *GPV* sample preparation.
