# Supplementary material for: New Evidence of the Bidentate Binding Mode in 3-MBA Protected Gold Clusters: Analysis of Aqueous 13–18 kDa Gold-Thiolate Clusters by HPLC-ESI-MS Reveals Special Compositions Aun(3-MBA)p, (n = 48–67, p = 26–30)
Source: Nanomaterials (Basel). 2019 Sep 11;9(9):1303. doi: 10.3390/nano9091303 (PMC6781097; doi:10.3390/nano9091303)
Supplement: Supplementary file 1 [file nanomaterials-09-01303-s001.pdf]

nanomaterials

MDPI

Article

New Evidence of the Bidentate Binding Mode in 3-MBA Protected Gold Clusters: Analysis of Aqueous 13–18 kDa Gold-Thiolate Clusters by HPLC-ESI-MS Reveals Special Compositions $Au_n(3\text{-MBA})_p$, (n = 48–67, p = 26–30)

David M Black [1,†], M. Mozammel Hoque [1,†,*], Germán Placencia-Villa [1,2] and Robert L Whetten [1,‡,*]

[1] Department of Physics & Astronomy, University of Texas, San Antonio, TX 78249, USA; black.david48@yahoo.com (D.M.B.); germanpvilla@gmail.com (G.P.V.)
[2] Department of Biology, University of Texas, San Antonio, TX 78249, USA
* Correspondence: mohammad.hoque64@gmail.com (M.M.H.); whettenz60@gmail.com (R.L.W.)
† These authors contributed equally to this work.
‡ Present Address: Department of Applied Physics and Material Science, Northern Arizona University, Flagstaff, AZ 86011, USA

Received: 22 August 2019; Accepted: 9 September 2019; Published: date

Table of Contents

1. Figure S1. Fine-structure in the electrospray negative ionization mode mass spectrometric analysis of the (67, 30), complex $[67, 30]^{z-}$ in solution.
2. Figure S2. Electrospray positive ionization (ESI+) mass spectrometric analysis of the component identified as (67, 30), by HPLC-ESI-MS as in Figure 2.
3. Figure S3. ESI-MS Analysis of *GPV* sample preparation, under conditions wherein mainly the singly charged (z = 1-) ions are detected.
4. Figure S4. The polyacrylamide gel electrophoresis (PAGE) and HPLC analyses of *GPV* sample preparation.

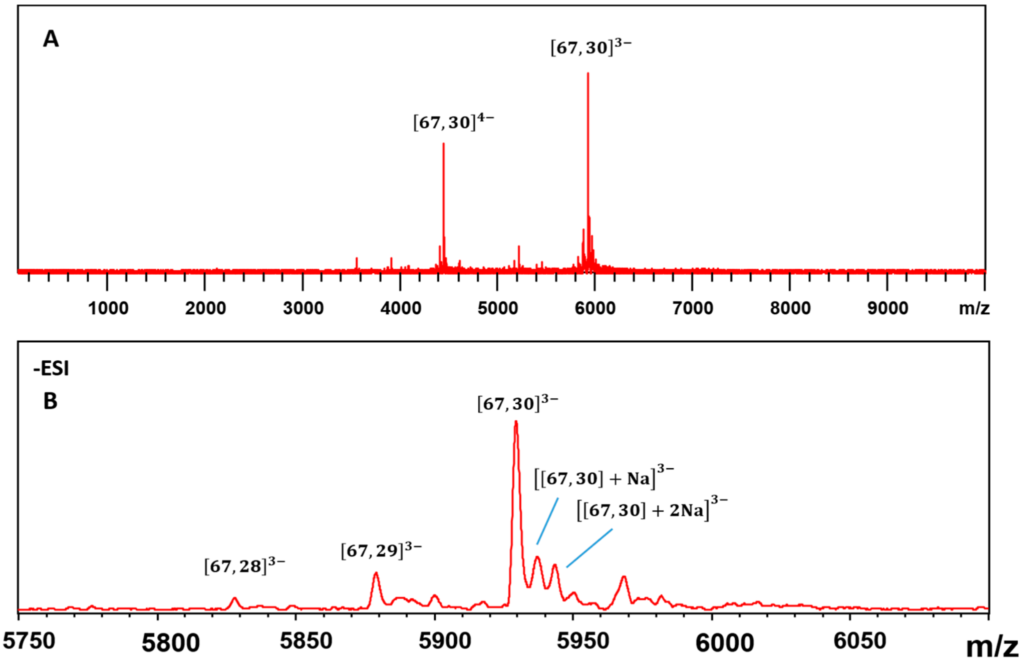

Figure S1. Fine-structure in the electrospray negative ionization mode mass spectrometric analysis of the (67, 30), complex (67, 30)$^{z-}$ in solution. (**A**) The cluster can be detected intact, thus confirming its composition and (3-, 4-) charge states. (**B**) The expansion of 3- charge state peak, showing proposed assignment of adduct and fragment peaks. Under these conditions, the cluster is induced to fragment by loss of ligands (30-29-28-27) while maintaining its Au-atom count fixed at 67. Several less dominant minor peaks are identified as sodium adducts.

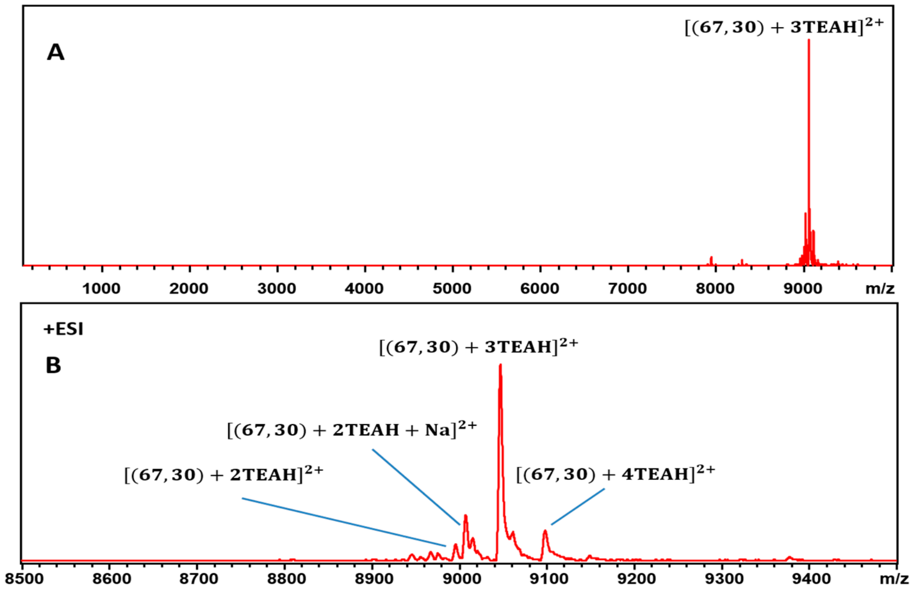

Figure S2. Electrospray positive ionization (ESI+) mass spectrometric analysis of the component identified as (67, 30), by HPLC-ESI-MS as in Figure 2. (**A**) The main peak is used to assign this composition, assuming 2+ charge state and three (3) triethylamine (TEA) adducts. (**B**) Close-up showing the assignment of features in the z = 2+ region. Note that other assignments preserve the (67, 30, *q*) label, where *q* designates the number of $TEAH^+$ and Na^+ adducts. The dominance of *q* = 3 peaks suggests the (67, 30) complex may have an intrinsic charge of (1^-), i.e. (67, 30)$^{1-}$, as discussed in the text. (However, note also that the mass of 3x TEAH = 306 and 2x MBA = 306 also, which one therefore cannot distinguish at the resolution shown.)

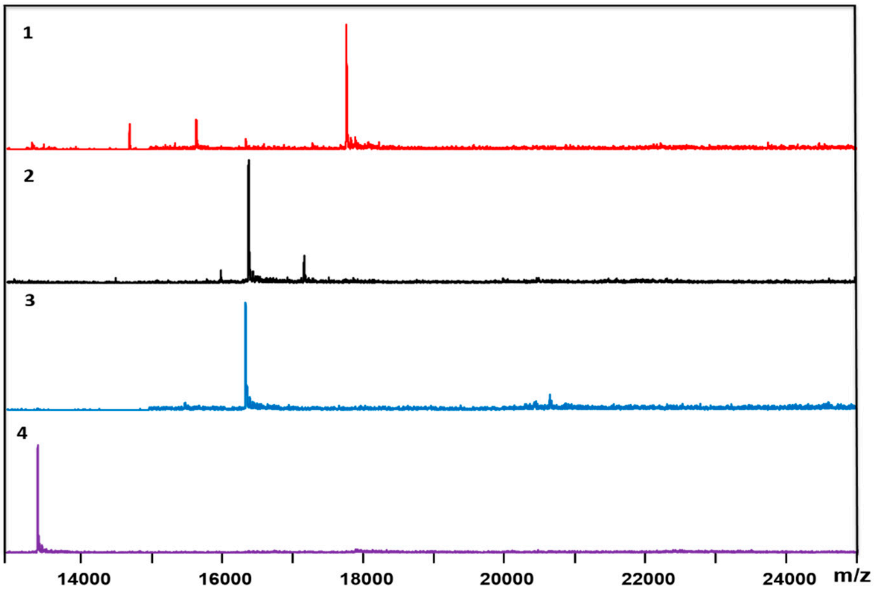

Figure S3. ESI-MS Analysis of GPV sample preparation, under conditions wherein mainly the singly charged (z = 1-) ions are detected. Negative ionization mode used for analysis. The color-coded chromatographic peaks track with the correspondingly coded and numbered for singly charged mass spectra listed here with proposed compositions: (1, Red) (67, 30), 17.8 kDa; (2, Black) (60, 31), 16.6 kDa; (3, Blue) (60, 30), 16.4 kDa; and (4, Purple) (48, 26), 13.4 kDa.

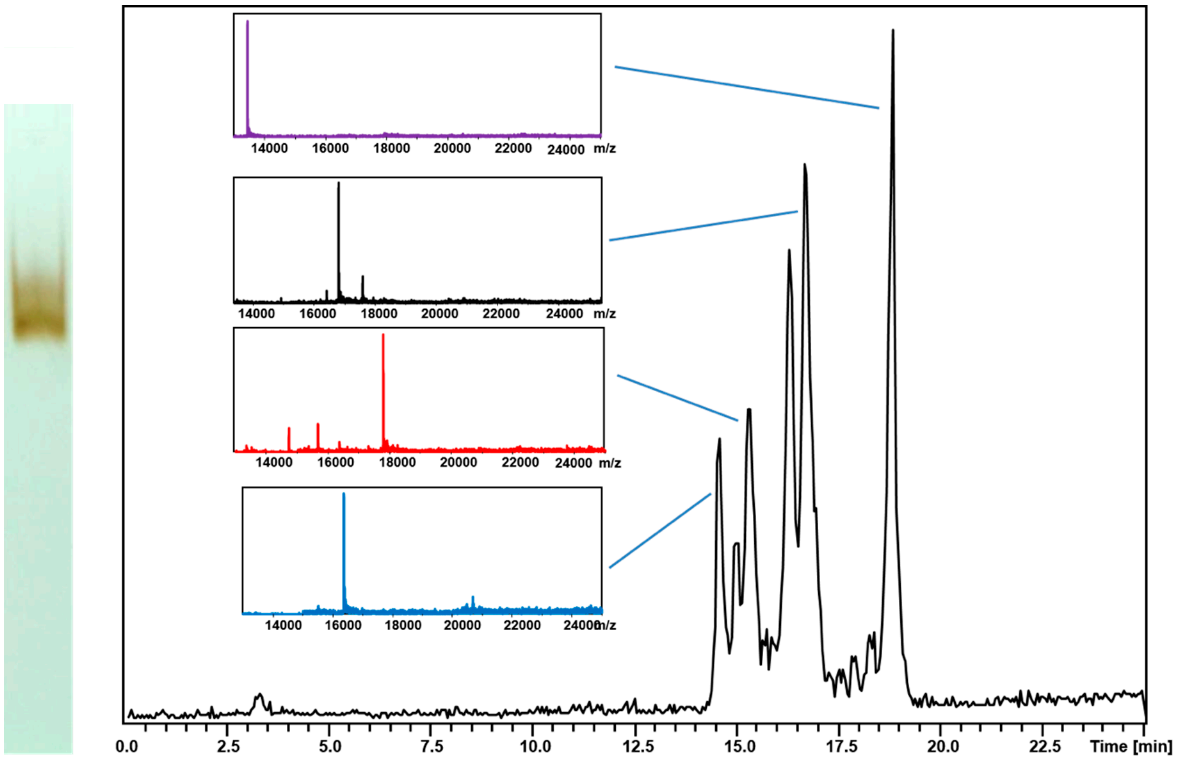

Figure S4. The polyacrylamide gel electrophoresis (PAGE) and HPLC analyses of *GPV* sample preparation. **(Left)** Photograph of the PAGE analysis image of the sample. (Right) HPLC chromatogram indicating correspondence to the mass spectra (inset) presented in Figure S3.

© 2019 by the authors. Submitted for possible open access publication under the terms and conditions of the Creative Commons Attribution (CC BY) license (http://creativecommons.org/licenses/by/4.0/).
